# Morphological Adaptations for Digging and Climate-Impacted Soil Properties Define Pocket Gopher (*Thomomys* spp.) Distributions

**DOI:** 10.1371/journal.pone.0064935

**Published:** 2013-05-24

**Authors:** Ariel E. Marcy, Scott Fendorf, James L. Patton, Elizabeth A. Hadly

**Affiliations:** 1 Department of Biology, Stanford University, Stanford, California, United States of America; 2 Department of Environmental Earth Systems Science, Stanford University, Stanford, California, United States of America; 3 Museum of Vertebrate Zoology, University of California, Berkeley, California, United States of America; Ecole Normale Supérieure de Lyon, France

## Abstract

Species ranges are mediated by physiology, environmental factors, and competition with other organisms. The allopatric distribution of five species of northern Californian pocket gophers (*Thomomys* spp.) is hypothesized to result from competitive exclusion. The five species in this environmentally heterogeneous region separate into two subgenera, *Thomomys* or *Megascapheus*, which have divergent digging styles. While all pocket gophers dig with their claws, the tooth-digging adaptations of subgenus *Megascapheus* allow access to harder soils and climate-protected depths. In a Northern Californian locality, replacement of subgenus *Thomomys* with subgenus *Megascapheus* occurred gradually during the Pleistocene-Holocene transition. Concurrent climate change over this transition suggests that environmental factors – in addition to soil – define pocket gopher distributional limits. Here we show 1) that all pocket gophers occupy the subset of less energetically costly soils and 2) that subgenera sort by percent soil clay, bulk density, and shrink-swell capacity (a mineralogical attribute). While clay and bulk density (without major perturbations) stay constant over decades to millennia, low precipitation and high temperatures can cause shrink-swell clays to crack and harden within days. The strong yet underappreciated interaction between soil and moisture on the distribution of vertebrates is rarely considered when projecting species responses to climatic change. Furthermore, increased precipitation alters the weathering processes that create shrink-swell minerals. Two projected outcomes of ongoing climate change—higher temperatures and precipitation—will dramatically impact hardness of soil with shrink-swell minerals. Current climate models do not include factors controlling soil hardness, despite its impact on all organisms that depend on a stable soil structure.

## Introduction

A large proportion of ecological work currently pursues how species have responded, or will respond, to environmental changes. This knowledge informs us of the past and may be necessary to avoid biodiversity collapse in the future [1]. Predicting one possible short-term response – geographic range shift – requires understanding how environmental factors, interspecific competition, and morphology maintain an organism's current range. Pocket gophers (family Geomyidae) provide an ideal study system because they 1) occupy the energetically demanding subterranean niche [2]; 2) exhibit variation in functional morphology directly related to exploitation of that niche [3]; and 3) different species distribute into neighboring, yet rarely overlapping ranges [4]. Interspecific differences in body size and digging strategy have been hypothesized to confer competitive dominance of one species over another depending on soil characteristics [3]–[6]. Understanding why pocket gopher species maintain these largely allopatric ranges could inform the effect of changing soil conditions on communities of subterranean organisms.

The subterranean niche requires animals to overcome humid environments with limited ventilation, low primary productivity, and the high energetic cost of foraging underground [7]. In response to such selection pressures, pocket gophers have acquired thermoregulatory and digging adaptations [7]. While we address thermoregulation, our study primarily highlights digging costs as the most informative factor for predicting pocket gopher range shifts. This view is consistent with thorough bioenergetic studies, which report that total energy balance depends on digging [5], [8]. Digging requires 360–3400 times more energy than walking the same distance [2]. A bioenergetics perspective would predict adaptations in pocket gopher behavior that reduce the cost of burrowing. For example, relative to body mass, mathematical models accurately predict tunnel diameter to be as small as possible [9]. This reduces both the cross-sectional area to shear and the volume of soil to displace [9], [10]. The exact digging cost, however, varies based on soil type and the digging strategy used to move the soil [2], [10], [11]. Pocket gopher morphology and burrow dimensions thus minimize the cost of foraging [9].

Species of western pocket gophers (genus *Thomomys*) inhabit a wide selection of soils across the environmentally heterogeneous North American west [4]. Furthermore, the two subgenera of genus *Thomomys*, *Thomomys* and *Megascapheus*, diverge in both body size and morphological adaptations for digging. Most notably, species in subgenus *Megascapheus* gained additional tooth-digging abilities through skull modifications and/or larger body size [3]. Teeth, the hardest material in the mammalian body, are rooted rigidly in the skull [3]; thus, tooth-digging confers an advantage through harder soils. In contrast, claw-digging remains the predominant strategy used by subgenus *Thomomys*. This method relies on softer keratinous claws mounted on relatively flexible digits, restricting this strategy to softer soils [3]. To be clear, these strategies are not diametric; all pocket gophers use claw-digging [3], [4] and even non-fossorial animals may make limited use of teeth for digging [12]. The acute angle of orthodont incisors in subgenus *Thomomys*, however, requires them to assume exaggerated head positions to use the working edge of their incisors for digging [4]. In contrast, the derived skull morphology of *Megascapheus* increases incisor procumbency to provide a mechanical advantage for tooth-digging without exaggerated head positions [4]. This enables animals to use their incisors to dig more effectively in hard soils and over extended periods of time [3], [4], [13]. Within each subgenus interspecific variation exists; however, overarching morphological differences allow us to use subgeneric distinctions as a proxy for predominant digging strategies.

When the unusually strict allopatry in genus *Thomomys* is discussed, correlations between morphological differences and soil type are often noted [4], [14]. In an extensive study of northeastern California, Thaeler observed that many species boundaries and limited cases of range overlap occur in regions with two divergent soil types [4]. In these regions, harder soils accommodated *Megascapheus* (two species: *bottae* and *townsendi*) and softer soils accommodated subgenus *Thomomys* (three species: *mazama*, *monticola*, and *talpoides*) [4]. Many studies cite tooth-digging as a *Megascapheus* advantage over the predominantly claw-digging subgenus *Thomomys* and genus *Geomys* pocket gophers in harder soils [3], [4]. Multiple studies show that tooth-digging species use less energy in harder soils and/or have higher burrowing rates than claw-digging counterparts [5], [11], [15]. Laboratory experiments on Geomyids demonstrated that predominantly claw-digging species soon abandoned efforts to dig in hard soil while a *Megascapheus* species, after attempting claw-digging, employed tooth-digging extensively [3]. A similar experiment in *Ctenomys*, a South American subterranean rodent similar to pocket gophers, corroborated these results [13]. Worldwide patterns of convergent evolution suggest that hard soils select for tooth-digging morphologies in subterranean rodents [6]. Tooth-digging species can occupy both hard and soft soils; however, bulky adaptations for tooth-digging may present a bioenergetics trade-off in soft soils because larger tunnel diameters require more excavation [9]. Thus, while each species can occupy a range of environments, each digging strategy appears to perform best under a particular combination of soil and climatic conditions [4].

**Figure 1:**
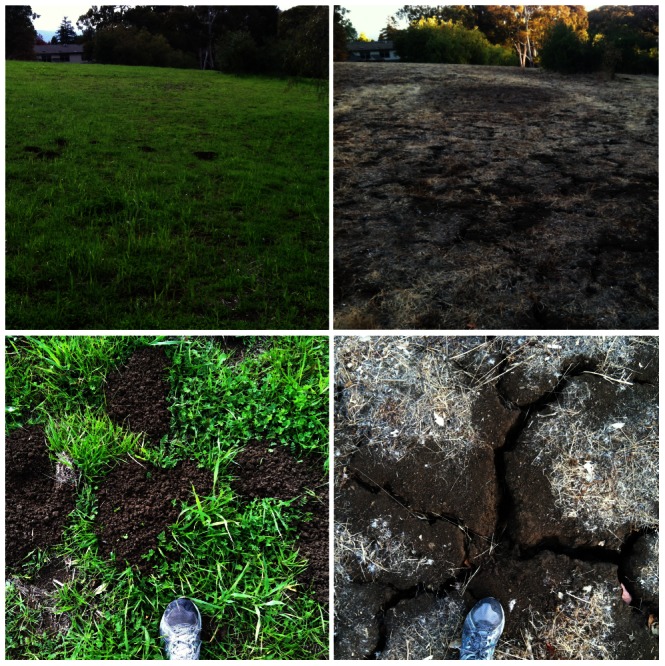
Linear extensibility affects pocket gopher activity on Stanford campus. The soils in the area photographed have a linear extensibility of 4.5%. A and B depict the area during the rainy winter. Fresh signs of pocket gopher activity are visible. C and D depict the same area with hardened and cracked soils during the arid Mediterranean summer; cracks of this type can reach up to 1 m deep [21]. *Megascapheus* pocket gophers inhabit this region of California, which is south of our study area. Photos by AEM.

Until now, the specific attributes qualifying soil as “soft” or “hard” with respect to pocket gophers were ill defined. Many studies cite relative values of percent clay (the part of soil texture that confers plasticity, and in high amounts, makes soil difficult to manipulate) or bulk density (an indicator of soil compaction calculated by the dry weight of soil divided by its volume) e.g., [14]. But these attributes are not necessarily correlated with soil hardness individually; soil hardness is better understood as a combination of clay and bulk density. For example, highly compactable clays produce the densest soils but sand is the heaviest particle size of soil; therefore a “soft” sandy soil could have a high bulk density [16]. The type of clay mineral presents a third, previously unspecified, attribute relevant to soil hardness. Soils enriched with smectite minerals, which are prevalent in California, expand when wet due to strong absorptive forces arising from an electrified interlayer within the clay structure and harden when dry due to adhesive forces [16]–[19]. Linear extensibility quantifies the shrink-swell capacity of soil. This property depends primarily on the amount of smectite clay present in the soil and is visible only when dry, warm climatic conditions reduce the effective moisture in the soil [19]. In California, the hot and dry Mediterranean summer causes smectite-enriched soils to shrink and harden [16], [17], impacting pocket gopher digging activity [20] (Fig. 1). High values of percent clay, bulk density, and linear extensibility, alone and especially all three combined, increase both shearing and displacement costs to pocket gophers moving soil [2], [9]. Furthermore, in response to a dry climate, linear extensible soils form cracks up to several meters deep and shift soil elevation on the order of centimeters [21]. This suddenly decreases plant availability and the structural integrity of burrows [21].

In addition to determining the energetic cost of digging, soil facilitates the majority of heat loss for pocket gophers [22]. Limited ventilation in the humid underground environment precludes evaporation and convection as viable physiological cooling mechanisms; therefore pocket gophers depend primarily on conduction through contact with the soil [7], [22]. Particularly important for dissipating metabolic heat during digging, the stable temperatures of deep underground burrows provide pocket gophers protection from thermal stress [7]. Stable temperatures exist at greater than 50cm [23], and at 1m depth, the soil temperature roughly equilibrates to the local annual average at the surface [16]. Thus the depth of soil available for burrow construction likely plays a role in pocket gopher distributions [4], [6]. Therefore, we hypothesize that pocket gopher distributions depend primarily on depth to bedrock, percent soil clay, bulk density, linear extensibility (a proxy for the clay mineral smectite), as well as the timing and amount of effective moisture.

Our study tests quantitatively whether each morphologically distinct pocket gopher subgenus associates with specific soils above or below physically significant soil threshold values. Previous investigations on soil and Geomyid distributions limit spatial scale, compare across genera, or use only one species, e.g., [24]–[26]. Often, only one aspect of soil is considered, e.g., texture but not bulk density nor linear extensibility. Here, we provide the first large-scale, quantitative analysis of how specific soil attributes best predict the biogeography of subgenera within a genus of pocket gophers. Our results show that 1) all genus *Thomomys* pocket gophers tend to occupy the subset of less energetically costly soils and 2) subgenera sort by percent soil clay, bulk density, depth to bedrock, and linear extensibility. The last attribute, a proxy for shrink-swell capacity, provides a mechanism by which climate can rapidly change the physical attributes of soil.

## Methods

**Figure 2:**
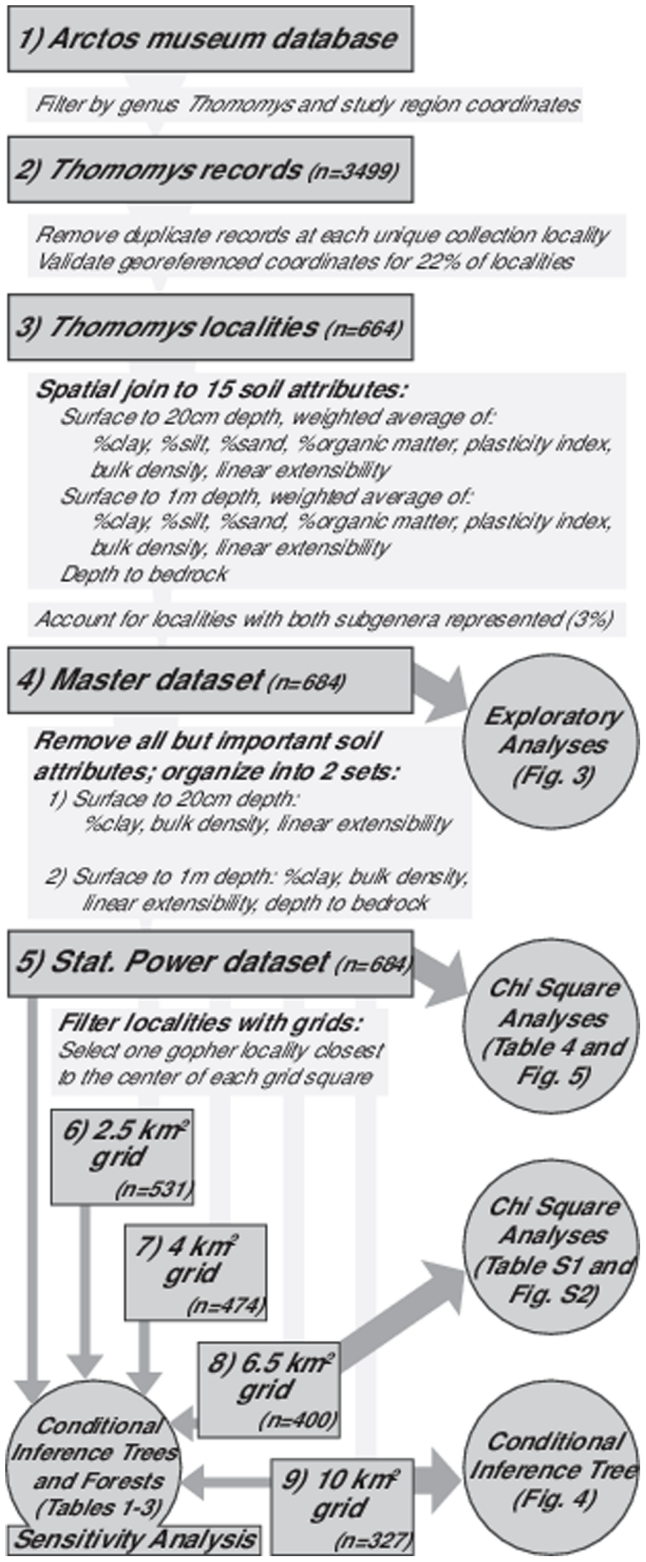
Methods summary. Flowchart outlining how we prepared the data (in rectangles) for our analyses (in circles). Dataset numbers are referred to in the text.

We used Geographic Information Systems (GIS) to join the rich, georeferenced datasets for genus *Thomomys* collection localities and for the soil of the northern California region. For all 664 localities, we extracted values of fifteen soil attributes with plausible impact on pocket gophers. Attributes were extracted from two different depths: 1) the depth containing foraging tunnels (surface to 20 cm), which constitute 60%–90% of the burrow system [2], [27], [28]; and 2) the depth containing the entire burrow system (surface to 1 m), which includes foraging tunnels as well as the deeper nests, food storage areas, and latrines [27], [29]. Exploratory analyses allowed us to test whether the soil attributes we expected to be important accounted for variation between species. Conditional inference tree analyses and Chi-squared tests used only these attributes to increase statistical power. Conditional inference tree analyses tested how multiple soil attributes split genus *Thomomys* subgenera into statistically significant categories of soil. Conditional inference forests ranked the importance of each predictor variable. Chi-squared tests analyzed the impact of each soil attribute separately on genus *Thomomys* distributions as well as tested whether subgenera split at attribute values with functional significance (e.g. soils with a percent clay below 20% fall into sandy and loam categories, soils easier to dig in). Methods are summarized in Figure 2.

### Data collection, verification, and processing

Genus *Thomomys* locality data came from Arctos, a multi-museum collection database. Soil data was extracted from the U.S. Department of Agriculture, National Resources Conservation Service (NRCS) STATSGO 2006 Digital General Soil Map of the United States. We extracted values for percent clay, silt, sand and organic matter; and indices of bulk density (oven dry at 1/3 bar water tension), plastic limit, and linear extensibility with the NRCS Soil Data Viewer extension to ArcMap 9.3.1 using weighted average aggregation from the soil surface to depths of 20 cm and 1 m. We also extracted the depth to bedrock and depth to the first restrictive layer. We clipped the *Thomomys* locality dataset and each of the soil attribute datasets to the study area defined by a rectangle created in geographic coordinate system WGS1994 with coordinates in decimal degrees (−124.494, 42.161) and (−119.035, 39.011). This region expands Thaeler's 1968 study area [4] to include specimens from nearby Nevada, Oregon, and northwestern California. We removed duplicate records for each unique collection locality.

The resulting dataset (#3 in Fig. 2) is composed of genus *Thomomys* specimens from the Museum of Vertebrate Zoology (MVZ) at University of California, Berkeley. Most specimens were georeferenced years after collection using the MaNIS protocol [30]. This protocol only uses the locality description to assign a coordinate and an error radius; it has become standard in retrospective georeferencing [30]. We conducted an extensive validation of locality accuracy by referring back to the original collector field notes from the MVZ archives and inputting all locality information into Geolocate, an online georeferencing tool for natural history data. To assess the average inaccuracy of our original dataset, we took a random sample of 40 pocket gopher localities from the dataset and checked them extensively against the field notes. 34 of the 40 records had notes available that described the collection location in greater detail than the locality description alone. On average the re-georeferrenced location was 1.3 km away from the original location.

Furthermore, to improve the quality of our dataset, we re-georeferenced an additional 112 records that had one or more red flags: coordinate uncertainty radii over 6.5 km, located in a lake or having “lake” in the locality description, coordinate precision less than three decimal places, and having the same coordinates as another distinct locality (this occurred occasionally with neighboring sites; e.g. 1.8 mi W, 0.1 mi N Beckwourth versus 1.9 mi W, 0.1 mi S Beckwourth). The records with uncertainty radii originally over 6.5 km moved on average 3.6 km, which reflects three outliers that placed old localities 74, 21, and 11 km away from our re-georeferenced sites (Fig. S1). The outliers were all products of typos and/or georeferencing mistakes in Township Range and Section information that placed the old coordinates kilometers away from the described locality. Preferential re-georeferencing of this set of “red-flagged” localities greatly increases our confidence in the validity of the dataset as a whole. We re-georeferenced 146 pocket gopher localities in total (22% of the dataset); the new coordinates have been reported to the MVZ to become part of their records. On average the distance between old and new locations was 1.8 km. This distance is less than the sensitivity of the NRCS STATSGO soil maps we used for our analyses, for which the approximate minimum area delineated is 6.25 square kilometers (1∶250,000 scale), a square with linear dimensions 2.5 km by 2.5 km.

In ESRI's ArcInfo GIS program (version 9.3.1), we converted all coordinate systems into GSC North American 1983. We transferred these data to ArcInfo version 10, in the USA Contiguous Equidistant Conic projection. For each pocket gopher locality, we recorded whether both subgenera were found at this locality (n = 20 localities or 3% of the dataset). We then joined the pocket gopher localities by location to soil attribute values for texture, organic matter, bulk density, plastic limit, and linear extensibility between the surface to 20 cm depth and the surface to 1 m depth, as well as the depth to bedrock and the depth to the shallowest restrictive layer. There were no instances in which depth to bedrock and depth to shallowest restrictive layer returned different values, so we removed the latter. In total, each of the pocket gopher localities was joined to 15 different soil attributes (dataset #4 in Fig. 2).

### Statistical Analyses

#### Exploratory analyses

We first analyzed separation of subgenera by soil texture using a soil texture triangle. For some locations, the textural parts of soil (percent sand, silt, and clay) did not sum to 100%. This is a natural outcome for interpolated data, but for this analysis we removed specimens with parts of soil summing to less than 80% or more than 120% for both depths. For all other points, we scaled the total particle size distribution to 100% while maintaining their proportions. These data were compared to the soil texture available in the study area.

We then analyzed separation of subgenera by all soil attributes with a Principal Components Analysis (PCA). The Joliffe cut-off value for each principal component eigenvector determines the number of significant principal components that should be considered. We used this data reduction process to choose the smallest number of explanatory soil attributes, increasing the statistical power of the conditional inference tree analyses.

#### Subgenera separation by nested soil attributes and sensitivity analysis

Conditional inference tree analysis uses multiple predictor attributes to recursively separate subgenera into mutually exclusive groups. Similar to decision trees, which were recently touted for ecological data [31], conditional inference trees offer several statistical improvements [32]. We used the *party* package in R to build ctrees which accommodate predictor variables with non-parametric distributions, control for covariance of predictor variables, and presents only statistically significant splits of the response variable to prevent over-fitting [32]. We used two sets of predictor attributes to analyze subgenera over the surface to 20 cm depth and over the surface to 1 m depth (dataset #5 in Fig. 2).

Each conditional inference tree is one of many that could be produced from a subset of the data. Conditional inference forests made with cforest from the *party* package generate a large number of ctrees (n = 500) by bootstrapping the dataset and averaging observation weights from each tree. While similar to random forests, this method provides unbiased variable selection in each tree and uses bootstrap sampling without replacement [33]. The resulting variable importance measures can be used to straightforwardly rank predictor variables even when they vary in their scale of measurement [33].

To address concerns that our pocket gopher dataset reflected sampling bias towards certain regions with more trapping effort from collectors, we conducted a sensitivity analysis on the conditional inference trees and forests. Using a series of four grids with increasing grid square dimensions (2.5 by 2.5 km, 4 by 4 km, 6.5 by 6.5 km, and 10 by 10 km) we selected one locality closest to the center of each grid square (datasets #6-9 in Fig. 2). We then compared the conditional inference trees and forests from all pocket gopher localities (n = 684), localities in a 2.5 km grid (n = 531), 4 km (n = 474), 6.5 km (n = 400), and 10 km grid (n = 327); see datasets #5-9 in Figure 2.

#### Chi-squared analyses of soil influence on and separation of pocket gopher subgenera

To complement the nested approach of conditional inference trees, Chi-squared analyses test the impact of each soil attribute separately on pocket gopher distributions. Furthermore, they test whether the subgenera split at soil attribute values with functional significance. Using dataset #5 (Fig. 2), we ran a series of Chi-squared tests with a null hypothesis of an even, random distribution of pocket gophers across the study area. We calculated the expected number of pocket gopher localities for each soil attribute by multiplying the proportion of the study area in each soil bin by the number of pocket gopher localities for each subgenus.

To assess how each soil attribute affects the distribution of pocket gophers, we focused on three sets of Chi-squared values. 1) Soil bin Chi-squared values compare the expected and observed values for one subgenus in one soil bin. We could not test for significant differences in a one by one Chi-squared table; however, values above 3.50 reflect a significant portion of the 5.99 critical value (CV) required for significance in the subgenus Chi square test. CV above 3.50 suggests a trend for over- or under-representation of a subgenus in a particular soil bin relative to chance. 2) The subgenus Chi-squared tests encompass all three soil bins for one subgenus. The magnitude indicates the strength of the soil attribute's influence on members in that subgenus. With these tests, we compared the relative influence of a soil attribute on subgenus *Thomomys* versus subgenus *Megascapheus*. With two degrees of freedom, critical numbers can be found to determine whether the soil influence is statistically significant (df = 2; p = 0.05, CV = 5.99; p = 0.01, CV = 9.21; p = 0.001, CV = 13.82). 3) The genus Chi-squared test combines Chi-squared values from both subgenera to indicate the attribute's overall influence on genus *Thomomys* (df = 4; p = 0.05, CV = 9.49; p = 0.01, CV = 13.28; p = 0.001, CV = 18.47).

## Results

### Exploratory analyses of soil attributes

**Figure 3:**
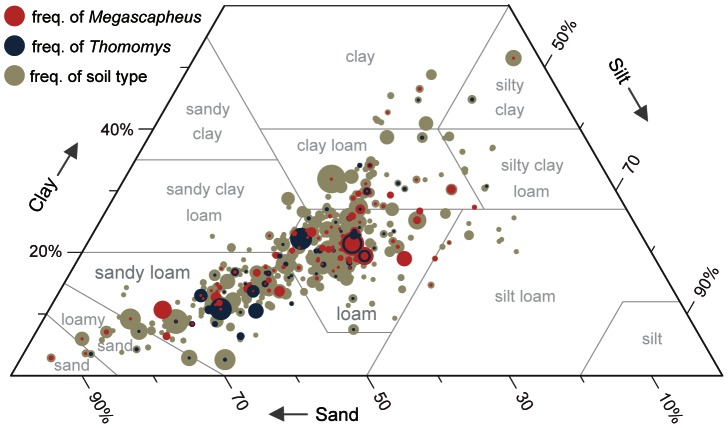
Genus *Thomomys* prefer sandy-loam and loam soils. Genus *Thomomys* subgenera plotted by soil texture. Red circles indicate soil types inhabited by *Megascapheus*, the subgenus with additional tooth-digging adaptations. Blue circles indicate soil types inhabited by the predominantly claw-digging subgenus *Thomomys*. Circle size indicates the frequency of pocket gopher subgenera or the frequency of soil types available in the study region.

The pocket gopher distribution across soil textures (Fig. 3) corroborates a preference reported in the literature for sandy-loam and loam soils [34]. Pocket gophers access the entire range of silt and sand contents in soils of the study area; however, clay content above 30% excludes most pocket gophers. These data show that pocket gophers occupy the subset of ideal burrowing soils available. While *Megascapheus* tend to occupy soils with higher clay content, texture alone cannot predict range boundaries between species (Fig. 3).

To identify additional soil attributes that could better account for range boundaries, we analyzed seven soil attribute values from two depths as well as total depth available in a Principal Component Analysis. The Joliffe cut-off removed all but three principal components, which accounted for 87.2% of the variance. As expected, the most informative attributes are clay and linear extensibility (a product of clay mineralogy), bulk density, and depth to bedrock.

### Subgenera separation by nested soil attributes and sensitivity analysis

**Figure 4:**
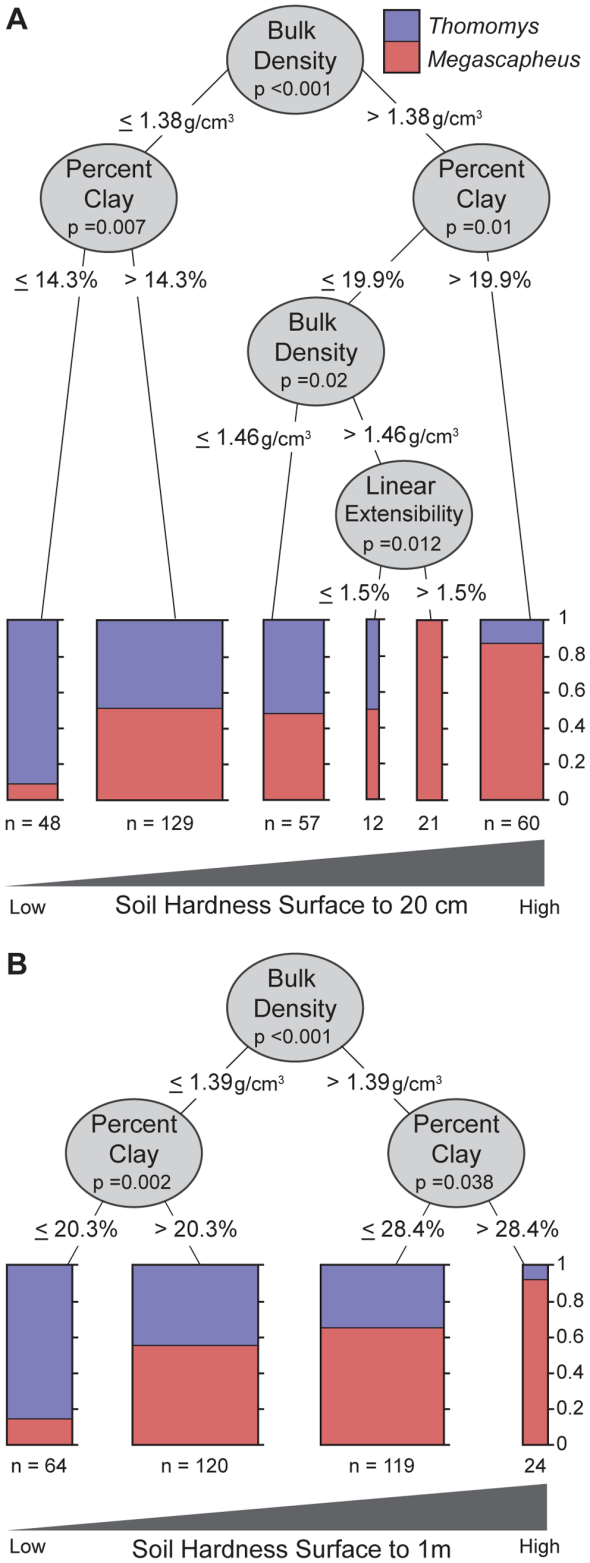
Combinations of soil attributes sort *Megascapheus* into harder soils; subgenus *Thomomys* into softer soils. Conditional inference trees from the 10 km grid dataset (n = 327). A is of soil attributes extracted over the surface to 20 cm depth; B is of soil attributes extracted over the surface to 1 m depth. The 10 km grid datasets are representative of all the datasets in the sensitivity analysis. Each node represents a split based on a critical value for one soil attribute. The p-value quantifies the degree of certainty by which this node improves the separation of the two subgenera. Branches to the left include pocket gophers that are found in soils below the critical value; branches to the right include pocket gophers that are found in soils above the critical value. This process continues iteratively for each branch until no more statistically significant splits can be made. The resulting plots show the proportion of pocket gophers of each subgenus found at the end of each branch. Red represents subgenus *Megascapheus*; blue represents subgenus *Thomomys*. The width of the plot represents the number of pocket gophers at the end of each branch. Plots to the left represent pocket gophers found in softer soils; plots to the right represent pocket gophers found in harder soils.

The conditional inference trees demonstrate that subgenus *Megascapheus* associates with more energetically demanding soil types while subgenus *Thomomys* associates with softer soils. This is true for soil at the foraging depth (surface to 20 cm) (Fig. 4A) and at the entire burrow depth (surface to 1 m) (Fig. 4B). Almost all of the branch splits in the conditional inference trees came very close to soil attribute thresholds we hypothesized *a priori* for their functional significance to digging (Table 1).

**Table 1:** Conditional inference tree critical values are similar to physically significant soil thresholds.

Soil Attribute	Conditional Inference Tree Cut-offs	*A Priori* Threshold	Physical Significance
Bulk density	1.01, 1.15 g/cm^3^	1.1 g/cm^3^	Soils with BD less than 1.1 g/cm^3^, irregardless of soil texture, have high void-space volume and/or low-density solids (e.g. organic matter) [16]. These soils are both light and soft.
	1.36, 1.38, 1.39, 1.41, 1.42, 1.46 g/cm^3^	1.4 g/cm^3^	A value of 1.4 g/cm^3^ for BD represents the transition to soils universally having prohibitively high hardness values [18]. A BD this high can restrict water storage and root penetration [18].
Percent clay	18.3, 19.6, 19.9, 20.3, 21, 21.4%	20%	Soils having less than 20% clay are categorized as sandy to loamy textures, soft soils that are relatively easy to dig [18].
	25.8, 27%	30%	30% clay represents a threshold above which soils fall into the heavy clay-loam to clayey textures and is a major distinguishing feature of Vertisols [18]. Combined with linear extensible smectite minerals, high hardness develops [19].
Linear ext.	1.5%	1.5%	LE is a proxy for smectite clay content, an important determinant, combined with drying, for soil hardness [16].
	2.8%	3%	LE above 3% can cause structural damage to human infrastructure and plant roots [16].
Depth to bedrock	38 cm	50 cm	Temperatures below this depth are stable (i.e. almost constant) through the day and night cycle [16], [23].
	77 cm	1 m	Temperatures at this depth stay almost constant year-round; within +/−5°C the aboveground annual average for the region [16].

For each soil attribute, we list the conditional inference tree cut-offs from our sensitivity analysis, the majority of which came close to values with known physical significance for digging animals.

In our sensitivity analysis, the conditional inference forests for all five datasets over the surface to 20 cm depth consistently ranked soil attributes percent clay, bulk density, and linear extensibility in order of importance (Table 2). The conditional inference forests for all five datasets over the surface to 1 m depth also consistently ranked percent clay, bulk density, linear extensibility in the same order with depth to bedrock last (Table 3). In general, clay and bulk density had variable importance measures about three to four times that of linear extensibility and depth (Tables 2 and 3). These trends are seen over both depths for all five pocket gopher locality datasets (#5-9 in Fig. 2) in the sensitivity analysis (Table 2), suggesting that they are not a product of sampling bias. Furthermore, the decrease in variable importance as the separation between localities increases suggests that the distribution pattern we observe is most important over local scales – i.e. where soil type is biologically important. In other words, competitive exclusion based on soil type can only occur across the small distances juvenile pocket gophers disperse (on average 57–239 m depending on species with a range of 7.5–789 m) [28], [34], [35].

**Table 2:** Sensitivity analysis of conditional inference forest variable importance for foraging tunnel depth.

	Variable Importance				
Soil Attribute	No grid	2.5×2.5 km	4×4 km	6.5×6.5 km	10×10 km
Percent clay Surface-20 cm	0.117	0.100	0.113	0.117	0.096
Bulk density Surface-20 cm	0.114	0.093	0.097	0.084	0.075
Linear ext. Surface-20 cm	0.052	0.034	0.032	0.015	0.016

Using the three soil attributes averaged over the surface to 20 cm depth, we ran a conditional inference forest (n trees = 500) to rank the soil attributes by variable importance measures. This is an indicator of much each soil attribute contributes to the accuracy of the tree relative to other soil attributes.

**Table 3:** Sensitivity analysis of conditional inference forest variable importance for entire burrow depth.

	Variable Importance				
Soil Attribute	No grid	2.5×2.5 km	4×4 km	6.5×6.5 km	10×10 km
Percent clay Surface-1 m	0.099	0.087	0.086	0.095	0.088
Bulk density Surface-1 m	0.129	0.104	0.100	0.093	0.090
Linear ext. Surface-1 m	0.026	0.037	0.031	0.017	0.012
Depth to bedrock	0.008	0.027	0.017	0.014	0.011

Using the three soil attributes averaged over the surface to 1 m depth as well as depth to bedrock, we ran a conditional inference forest (n trees = 500) to rank the soil attributes by variable importance measures. This is an indicator of much each soil attribute contributes to the accuracy of the tree relative to other soil attributes.

### Chi-squared tests

The Chi-squared tests assess whether pocket gophers respond to the physically significant soil thresholds outlined in Table 1. We used the no grid dataset because this dataset maintains gopher records within the biologically important distances over which competitive exclusion could occur (n = 684). However we also report the Chi-squared tests for the 6.5 km dataset, which show similar results (Table S1 and Fig. S2).

**Figure 5:**
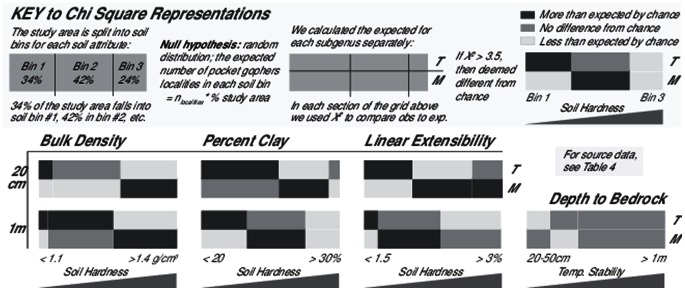
*Megascapheus* inhabit harder soils more often than expected by chance; subgenus *Thomomys* show opposite trend. Null hypothesis for the Chi square tests: random distribution across the study area (n = 684). Expected values are proportional to the percent study area in each soil bin. For bulk density, percent clay, and linear extensibility at both depths, *Megascapheus* are found in harder soils more often than expected by chance and in softer soils less often than expected. Subgenus *Thomomys* show the opposite pattern: they are found in harder soils less often than expected and in softer soils more often than expected. Depth to bedrock does not produce as striking results; however, *Thomomys* are found in shallow soils less often than expected by chance.

Almost all of the subgenus and genus Chi-squared tests returned highly significant p values (p<0.01 or p<0.001). This demonstrates that genus *Thomomys* pocket gophers distribute non-randomly with respect to bulk density (Surface to 20 cm: χ^2^ = 81.15, df = 4, p<0.001; Surface to 1 m: χ^2^ = 91.07, df = 4, p<0.001), percent soil clay (Surface to 20 cm: χ^2^ = 28.50, df = 4, p<0.001; Surface to 1 m: χ^2^ = 65.93, df = 4, p<0.001), linear extensibility (Surface to 20 cm: χ^2^ = 38.74, df = 4, p<0.001; Surface to 1 m: χ^2^ = 70.25, df = 4, p<0.001), and depth to bedrock (χ^2^ = 17.93, df = 4, p <0.01). Soil bin Chi-squared values suggest that pocket gopher subgenera trend towards divergent soil types (Fig. 5). Corroborating the conditional inference tree analyses, subgenus *Thomomys* gophers skew towards low clay, low bulk density, and low linear extensible soils while *Megascapheus* gophers skew towards soil bins with higher attribute values (Fig. 5).

In general, soil attributes exert greater influence on the predominantly claw-digging subgenus *Thomomys* than on *Megascapheus*, the subgenus with additional tooth-digging adaptations, as suggested by the magnitude of Chi-square values (Table 4). For percent clay, bulk density, and linear extensibility, the genus Chi-squared test values over the surface to 1 m depth are higher than those over the surface to 20 cm depth. This trend is driven entirely by the increase in subgenus *Thomomys* Chi square values (Table 4). This trend occurs even while the subgenus *Megascapheus* Chi squares all decrease when comparing the 20 cm depth to the 1 m depth (Table 4). For each soil attribute over the surface to 1 m depth, the subgenus *Thomomys* Chi square values in the lowest and highest soil bins are around twice the Chi square value in the corresponding surface to 20 cm bins. This indicates that subgenus *Thomomys* skew more sharply from chance at the entire burrow depth than over the 20 cm depth. For example, almost 20% of subgenus *Thomomys* are found in the 10% of study area with extremely low linear extensible soils over the 1 m depth (Table 4).

**Table 4:** Soil bin, subgenus and genus Chi square results.

BULK DENSITY 20 cm								
*Soil Bin*	*Area (Km^2^)*	*% Area*	*Exp T.*	*Obs T.*	*Thom. X^2^*	*Exp M.*	*Obs M.*	*Meg. X^2^*
<1.1 g/cm^3^	1.5 E+10	0.10	34	64	26.38	36	13	14.95
1.1–1.4	7.3 E+10	0.49	162	165	0.05	173	137	7.43
>1.4	6.1 E+10	0.41	135	102	8.02	144	203	24.31
***Total***	1.5 E+11	1	331	331	34.46***	353	353	46.69***
						**Genus ** ***X^2^*** **:**		81.15***

BULK DENSITY 1 m								
*Soil Bin*	*Area (Km^2^)*	*% Area*	*Exp T.*	*Obs T.*	*Thom. X^2^*	*Exp M.*	*Obs M.*	*Meg. X^2^*
<1.1 g/cm^3^	1.1 E+10	0.07	24	56	42.75	26	5	16.55
1.1–1.4	6.9 E+10	0.47	154	178	3.76	164	152	0.90
>1.4	6.9 E+10	0.46	153	97	20.54	163	196	6.57
***Total***	1.5 E+11	1	331	331	67.04***	353	353	24.03***
						**Genus ** ***X^2^*** **:**		91.07***

PERCENT CLAY 20 cm								
*Soil Bin*	*Area (Km^2^)*	*% Area*	*Exp T.*	*Obs T.*	*Thom. X^2^*	*Exp M.*	*Obs M.*	*Meg. X^2^*
<20%	8.3 E+10	0.56	184	221	7.42	196	178	1.70
20–30%	5.4 E+10	0.36	120	89	8.23	129	157	6.32
>30%	1.2 E+10	0.08	26	21	1.13	28	18	3.70
***Total***	1.5 E+11	1	331	331	16.78***	353	353	11.72**
						**Genus ** ***X^2^*** **:**		28.50***

PERCENT CLAY 1 m								
*Soil Bin*	*Area (Km^2^)*	*% Area*	*Exp T.*	*Obs T.*	*Thom. X^2^*	*Exp M.*	*Obs M.*	*Meg. X^2^*
<20%	5.0 E+10	0.33	111	166	27.52	118	100	2.79
20–30%	6.3 E+10	0.42	140	129	0.79	149	182	7.42
>30%	3.6 E+10	0.24	81	36	24.77	86	71	2.64
***Total***	1.5 E+11	1	331	331	53.08***	353	353	12.85**
						**Genus ** ***X^2^*** **:**		65.93***

LINEAR EXTENSIBILITY 20 cm								
*Soil Bin*	*Area (Km^2^)*	*% Area*	*Exp T.*	*Obs T.*	*Thom. X^2^*	*Exp M.*	*Obs M.*	*Meg. X^2^*
<1.5%	5.3 E+10	0.35	117	147	7.63	125	69	25.01
1.5–3%	6.4 E+10	0.43	142	119	3.78	152	190	9.70
>3%	3.2 E+10	0.22	72	65	0.63	76	94	4.02
***Total***	1.5 E+11	1	331	331	12.04**	353	353	38.74***
						**Genus ** ***X^2^*** **:**		50.77***

LINEAR EXTENSIBILITY 1 m								
*Soil Bin*	*Area (Km^2^)*	*% Area*	*Exp T.*	*Obs T.*	*Thom. X^2^*	*Exp M.*	*Obs M.*	*Meg. X^2^*
<1.5%	1.5 E+10	0.10	35	64	25.13	37	13	15.42
1.5–3%	6.8 E+10	0.45	151	171	2.76	161	198	8.71
>3%	6.6 E+10	0.44	146	96	17.05	156	142	1.18
***Total***	1.5 E+11	1.00	331	331	44.94***	353	353	25.31***
						**Genus ** ***X^2^*** **:**		70.25***

DEPTH TO BEDROCK								
*Soil Bin*	*Area (Km^2^)*	*% Area*	*Exp T.*	*Obs T.*	*Thom. X^2^*	*Exp M.*	*Obs M.*	*Meg. X^2^*
20–50 cm	2.6 E+10	0.17	52	37	4.18	58	59	0.01
50-1 m	3.0 E+10	0.20	60	55	0.36	67	43	8.81
>1 m	9.6 E+10	0.63	191	210	1.96	215	239	2.61
***Total***	1.5 E+11	1.00	302	302	6.50*	341	341	11.43**
						**Genus ** ***X^2^*** **:**		17.93**

Soil bin Chi-squared values (in regular font) compare the expected and observed values for one subgenus in one soil bin. Values above 3.50 reflect a significant portion of the 5.99 critical value (CV) required for significance in the subgenus Chi square test. The subgenus Chi-squared tests (in bold) encompass all three soil bins for one subgenus (df = 2; p = 0.05*, CV = 5.99; p = 0.01**, CV = 9.21; p = 0.001***, CV = 13.82). The genus Chi-squared test combines Chi-squared values from both subgenera to indicate the attribute's overall influence on genus *Thomomys* (df = 4; p = 0.05*, CV = 9.49; p = 0.01**, CV = 13.28; p = 0.001***, CV = 18.47).

Percent clay, linear extensibility, and bulk density all increase at lower depths. The increase in clay content and soil structural unit size results in an increase in bulk density within the subsurface soil horizons [16]. Clay particles translocate to deeper depths via water, which is accelerated by pocket gophers digging [36]; therefore clay content, along with linear extensibility and bulk density, tends to increase with soil depth. Thus, the subset of accessible soils for subgenus *Thomomys* decreases with depth. Furthermore, subgenus *Thomomys* is underrepresented in soils with the shallowest depths with the least stable temperatures (20–50 cm); this trend drives most of the significant subgenus *Thomomys* Chi Square test for depth to bedrock (χ^2^ = 6.50, df = 2, p<0.05).

## Discussion

The characteristics of soils that confer difficulty in digging significantly separate the subgenera of genus *Thomomys* into different soil types. This is consistent with the existing literature, which attributes competitive exclusion to soil “hardness” [3], [4]. Our results, however, consider both percent soil clay and bulk density more precisely as the soil attributes that confer hardness. Furthermore, we introduce linear extensibility (related to clay mineral type, and specifically the presence of smectite clays) and depth to bedrock as important discriminating factors for the ranges of soil-dependent organisms. Before we discuss our findings further, we put in context two factors besides soil that concern pocket gopher distributions.

The first factor, plants, is often cited as the most important determinant of mammalian herbivores distributions, e.g., [37]. Bioenergetics studies on genus *Thomomys* report plant density – but not specific plant distributions – as an important factor, since it impacts how many hours a pocket gopher must dig in order to balance its energy budget [5]. Given the constraints of underground foraging, evolutionary ecologists argue that natural selection would favor generalists over specialists [6], [38]. Geomyids have been shown to eat a wide variety of above- and belowground plant parts, leaves, bulbs, tubers, woody plants, legumes, cultivated crops, roots, bark, acorns, and even fungi [6], [29], [35], [39]. In cafeteria-style experiments, all three subgenus *Thomomys* species (*mazama*, *monticola*, and *talpoides*) preferred plants with higher nutritional quality and moisture content [38], [40]. Stomach content analyses of all three subgenus *Thomomys* species and *T. (Megascapheus) bottae* reveal that their diets, while biased towards forbs over grasses, closely track the resources available seasonally [5], [6], [35], [41], [42]. The remaining *Megascapheus* species, *T. (M.) townsendii*, less well studied and restricted to a relatively smaller region, is the only species in our study group with some distributions purportedly dependent on a particular type of salt grass [43]. However, the correlation with salt grass does not hold for the entire distribution, and the species appears to be more dependent on the deep soil conditions found throughout its range [4].

The above literature suggests that preferred plant species affect pocket gopher habitat selections, but because there are few if any interspecific preference differences, it cannot account for the allopatric pattern of distribution. By many accounts, genus *Thomomys* pocket gophers share almost identical modes of life, similar food preferences, and likely all prefer deep, soft soils [5], [34], [41]. Like aboveground herbivores, however, when niches overlap competitive exclusion tends to occur e.g., [37]. For pocket gophers, the literature suggests aggressive behavior and dispersal ability as the most likely mechanisms [41], [44]. While agonistic behavior characterizes most interactions between individual pocket gophers [4], [27], data on aggression between species from the lab and the field are mixed e.g., [34] vs. [44] and have so far failed to explain genus *Thomomys* distributions [34], [44]. When an experimental field study introduced individuals of *T. (M.) bottae* and *T. (T.) talpoides* into novel areas, the ability to disperse – as impacted by reproduction and immigration rates – determined the success of one species over the other [34]. While dispersal tends to occur aboveground [45], reproduction rate certainly depends on access to nutritional food underground [5]. Factors influencing the rate of food acquisition include soil conditions and the consequent burrowing rate; a study on *T. (T.) talpoides* found that the highest adult mortality occurred during the harshest burrowing conditions [5]. A bioenergetics analysis revealed that nutritional plant density and burrowing rate precluded lactation and therefore occupancy for *T. (T.) talpoides* in soils otherwise within their abilities [5]. Given an area with a certain plant density and two competing pocket gophers, burrowing efficiency most likely determines the dominant species [5].

**Figure 6:**
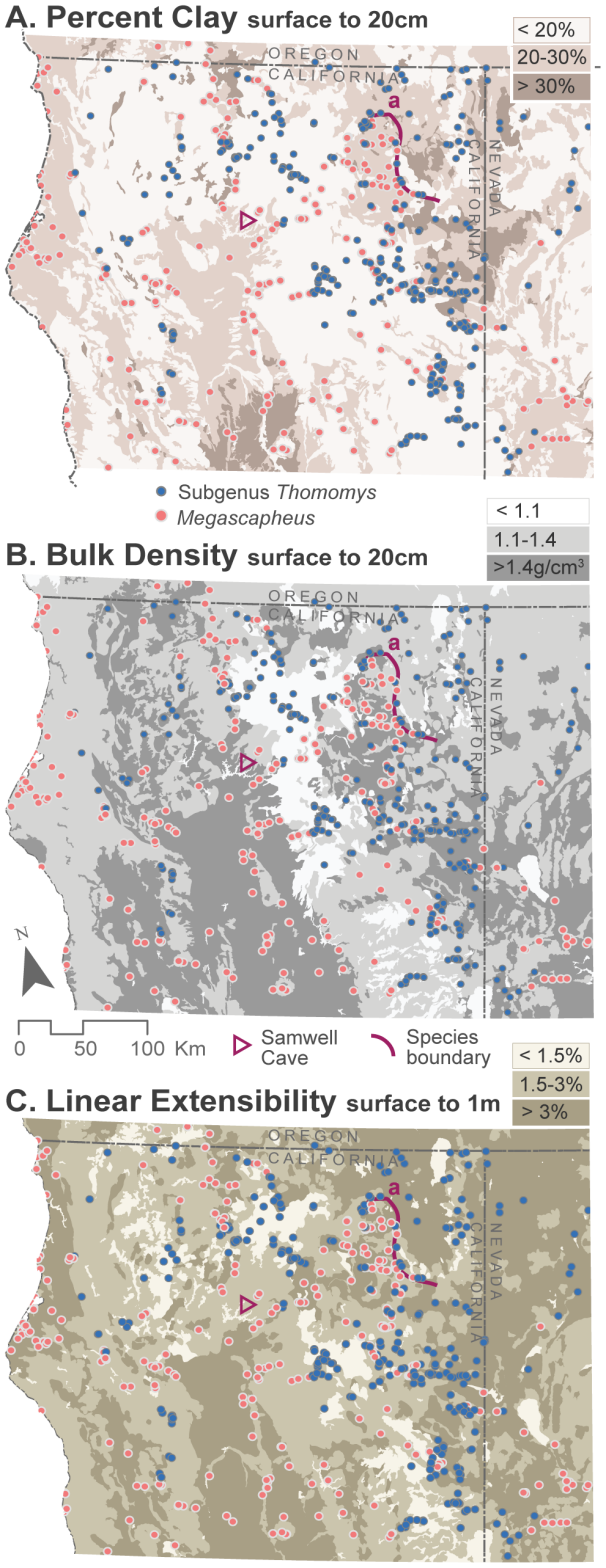
Changes in available moisture, and its impact on linear extensible soils, affect species boundaries. In the absence of severe human disturbance, percent clay and bulk density change on time scales of decades to millennia [16]. Hardness of a soil with high linear extensibility, however, can change in just days [19]. The triangle indicates the location of Samwell Cave, an area that records the presence of subgenus *Thomomys* during the cooler, wetter Pleistocene [47]. The boundary between subgenus *Thomomys* and *Megascapheus* appears to have shifted north over the transition to the Holocene, as the climate in the central valley became Mediterranean. Species boundary “a” is currently in an area of California that has cooler, wetter, continental summers in contrast to the central valley. If this were to change, we would expect the *Megascapheus* range to expand and the subgenus *Thomomys* range to contract.

The second factor, pre-emptive occupancy, refers to the biogeographic influence on modern distributions of pocket gophers independent of competitive exclusion – i.e. species occupy the regions they migrated to first. Historically, pocket gopher researchers have debated the relative importance of competitive exclusion and pre-emptive occupancy e.g., [4], [41]. As one field study shows, a nearby population increased dispersal via immigration into a contested area, providing an advantage to the pre-emptive species [34]. A more recent genetic analysis demonstrated that *T. (M.) bottae* have a long history in central California but populations only recently spread through much of our study area in northern California [46]. Consistent with modern genetics information, ancient DNA from a bone assemblage in Samwell Cave near Lake Shasta records a gradual replacement of *T. (T.) mazama* by *T. (M.) bottae* during the Pleistocene-Holocene transition [47] (Fig. 6C). This case and more contemporary unidirectional boundary movements [48] appear to be caused by changing environmental conditions affecting the soil and therefore operate via competitive exclusion. Certainly, explanations of current distributions and predictions of range shifts must take into account recruitment from existing populations [49]. Thus given historical distributions in northern California, we would expect to find instances of *Megascapheus* enjoying soft soils and subgenus *Thomomys* surviving in harder soils - but only in uncontested areas.

The evidence for competitive exclusion in pocket gophers includes functional morphological differences shown to provide dispersal advantages in particular soils as well as recent, unidirectional movements of species boundaries. Since our data demonstrates that subgenus *Megascapheus* can access a wider range of soil types compared to subgenus *Thomomys*, this suggests, but does not demand, that subgenus *Thomomys* exclude the former from more friable soils. Previous pocket gopher literature suggests that increased dispersal ability is a better predictor than behavioral dominance of which subgenus will prevail in a given area [5], [34], [44]. Our results suggest two possible mechanisms consistent with the dispersal hypothesis: 1) subgenus *Thomomys* are more energetically efficient in softer soils by requiring smaller tunnels or by requiring less energy to support a smaller body size [6]. 2) Tooth-digging adaptations allow *Megascapheus* to access harder and deeper soils, which are protected from changes caused by the ambient temperature and precipitation.

The importance of linear extensibility in the conditional inference trees and forests as well as the significance of the Chi-squared tests suggests that temperature and precipitation mediate pocket gopher distributions by modifying soil properties. Smectite clays associated with linear extensibility increase soil hardness in response to decreased water content of soils, or loss of effective moisture over time [16]–[19]. Considerations of linear extensibility are particularly pertinent in California where arid Mediterranean summers elicit dramatic responses from the region's high abundance of expandable clay minerals [17], [18]. In the absence of severe human disturbance, clay mineral content and bulk density change on time scales of decades to millennia [16]. Hardness of a soil with high linear extensibility, however, can change in just days [19]. To our knowledge, shrink-swell capacity has never been discussed as a soil attribute that determines pocket gopher ranges. By impeding excavation and damaging existing burrows, the cracking and hardening of soil has profound implications for any subterranean animal – and give larger, tooth-digging species an advantage.

Burrowing ecology studies show that climate affects how and when pocket gophers dig. In northern California, Bottae's pocket gopher, *T. (M.) bottae* showed preference for active mound building and burrow excavation from November through May, with almost no activity in the arid summer months of July, August and September [50]. Soil moisture appears to encourage digging, both as a response to workable soil and plant growth [15]. And while California pocket gophers typically aestivate, or become significantly less active in the summer [24], pocket gophers in irrigated fields continue to dig in the summer [51].

Evidence from ancient DNA suggests that pocket gopher distributions have been affected by climatic changes in the past [47]. During the Holocene-Pleistocene transition, aridification associated with regions at a fossil site near Mount Shasta (Samwell Cave) preserved evidence that a tooth-digging pocket gopher, *T. (M.) bottae*, gradually replaced a claw-digging pocket gopher, *T. (T.)* cf. *mazama*, which contracted its range to the north [47]. We hypothesize that changes in hardness resulting from desiccation of *in situ* smectite minerals with the development of more arid summers drove this turnover event. Elsewhere in the arid west, another claw-digging species, *T. (T.) talpoides* was also extirpated in low elevation valleys and replaced by the tooth-digging *T. (M.) bottae* [52]. Species in subgenus *Thomomys* could still inhabit linear extensible soils if soil moisture levels remain high enough for long enough to prevent shrink-swell activity. In the northeast corner of our study area, subgenus *Thomomys* exclude *Megascapheus* from relatively high linear extensible soils (species boundary a, Fig. 6C). This boundary, however, also coincides with a transition from hot and dry Mediterranean summers, to cooler, damper continental summers. The latter condition likely maintains higher soil moistures and prevents hardness resulting from desiccation of shrink-swell minerals. Should this climatic boundary shift, we predict that *Megascapheus* would displace subgenus *Thomomys* from soils above 3% LE and that soils with LE above 6% would exclude all pocket gophers.

The long-term soil response to climate change will depend on a number of factors including soil mineralogy, temperature, precipitation, and the time period under which these processes change the soil. Warmer, drier conditions will, in general, lead to a hardening of smectite dominated soils [17]. Further, long-term aridity could fundamentally change soil composition, leading to an additional hardening through carbonate-precipitated layers [53]. However, pocket gopher activity generates high levels of disturbance that retards the calcification process [53], [54]. Climate change trends predict not only warming but also a redistribution of precipitation in California [55]. In areas with increased rainfall, weathering – accelerated by warmer temperatures – would lead to a long-term change in mineralogy from smectite to kaolin clays, and generally decrease soil hardness [17].

For thousands of years, the balance of soil and climate appears to delicately delineate boundaries between competitive species of pocket gophers. Pocket gopher distribution responses to ongoing climate change will have ramifications for the communities and ecosystems to which they belong. Subterranean herbivores occupy a unique position as both ecosystem engineers and keystone species in the trophic chain [56]–[58]. Because subterranean rodents create distinctive habitat patches that maintain grassland biodiversity, a recent review has called for increased management efforts to support their populations [56]. Their burrows provide escape tunnels, breeding grounds, and homes for insects, herpetological fauna, and other mammals and therefore a source of prey for most types of carnivores [56]. More indirectly, burrow and mound construction along with underground foraging increases soil nutrients and water infiltration into the soil [56]. This may eventually support more biomass, a purported mutualism with megaherbivores [56]. Pocket gophers are specifically credited with creating habitats for endangered species, such as the burrowing owl; inhibiting invasive grass establishment; providing direct inoculation of soil with mychorrhizae, a mutualistic fungi for many species of plants; and otherwise influencing community structure over weeks, years, centuries and millennia [57]–[60]. As “geomorphic” agents, pocket gophers affect soil conditions by mixing soil layers, reducing topsoil depth, and over long periods of time, creating distinctive topographic hillocks called mima mounds visible from the air [61]. In fact, paleontological studies suggest that changes in pocket gopher burrowing in response to ancient climate change destabilized sand hills in Nebraska [62].

Pocket gophers respond to climatic factors through the soil response to changes in available moisture. This impact on a relatively large vertebrate underscores the importance of the soil response to changes in available moisture for all soil dependent organisms, notably plants. Despite forecasts of radical changes in temperature and precipitation, no climate change models account for such changes. Particularly in regions with Mediterranean climates and shrink-swell clays, the soil response to available moisture should be included in conservation and agricultural outcomes given ongoing climate change.

## Supporting Information

Figure S1 **Impact of validation on pocket gopher locality georeferences.** Old localities (dark red circles) were designated using only the brief locality description as per the MaNIS protocol. New localities (blue and pink circles) were designated by AEM using the collector's original field notes. Our validation targeted localities that had red flags for coordinate accuracy (n = 146, 22% of the dataset). In all but a few cases the difference in location varied only slightly. On average localities moved 1.8 km, which is less than the sensitivity of the underlying soil layer, 2.5 km. The exceptions, noted by the red lines denoting the distance moved, were caused by typos during the georeferencing process for the old localities.
(TIF)

Figure S2 **The much smaller 6.5 km grid dataset shows similar Chi square results as the full dataset.** Compare to Figure 5. The null hypothesis for the Chi square tests: random distribution across the study area for the 6.5 km grid dataset (n = 200). Expected values are proportional to the percent study area in each soil bin. For bulk density, percent clay, and linear extensibility at least one depth, *Megascapheus* are found in harder soils more often than expected by chance and in softer soils less often than expected. Subgenus *Thomomys* show the opposite pattern: they are found in harder soils less often than expected and in softer soils more often than expected. Depth to bedrock does not produce significant results in this dataset.
(EPS)

Table S1 **Similar to** **Table 4** **but reporting the 6.5 km grid dataset.** Soil bin Chi-squared values (in regular font) compare the expected and observed values for one subgenus in one soil bin. Values above 3.50 reflect a significant portion of the 5.99 critical value (CV) required for significance in the subgenus Chi square test. The subgenus Chi-squared tests (in bold) encompass all three soil bins for one subgenus (df = 2; p = 0.05*, CV = 5.99; p = 0.01**, CV = 9.21; p = 0.001***, CV = 13.82). The genus Chi-squared test combines Chi-squared values from both subgenera to indicate the attribute's overall influence on genus *Thomomys* (df = 4; p = 0.05*, CV = 9.49; p = 0.01**, CV = 13.28; p = 0.001***, CV = 18.47).
(DOC)
