# Supplementary figures and images for: Morphological Adaptations for Digging and Climate-Impacted Soil Properties Define Pocket Gopher (Thomomys spp.) Distributions
Source: PLoS One. 2013 May 24;8(5):e64935. doi: 10.1371/journal.pone.0064935 (PMC3663803; doi:10.1371/journal.pone.0064935)

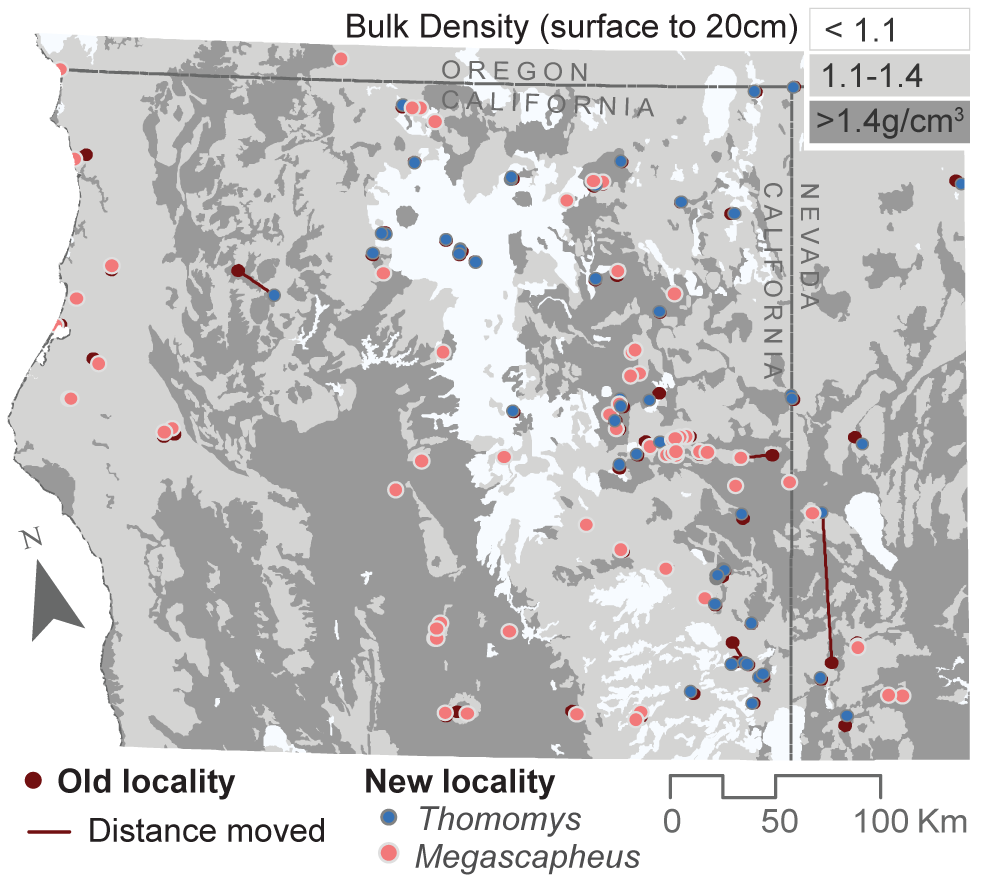

Supplement: Figure S1 — Impact of validation on pocket gopher locality georeferences. Old localities (dark red circles) were designated using only the brief locality description as per the MaNIS protocol. New localities (blue and pink circles) were designated by AEM using the collector's original field notes. Our validation targeted localities that had red flags for coordinate accuracy (n = 146, 22% of the dataset). In all but a few cases the difference in location varied only slightly. On average localities moved 1.8 km, which is less than the sensitivity of the underlying soil layer, 2.5 km. The exceptions, noted by the red lines denoting the distance moved, were caused by typos during the georeferencing process for the old localities. (TIF) [file pone.0064935.s001.tif]
