# Supplementary material for: Morphological Adaptations for Digging and Climate-Impacted Soil Properties Define Pocket Gopher (Thomomys spp.) Distributions
Source: PLoS One. 2013 May 24;8(5):e64935. doi: 10.1371/journal.pone.0064935 (PMC3663803; doi:10.1371/journal.pone.0064935)
Supplement: Table S1 — Similar to Table 4 but reporting the 6.5 km grid dataset. Soil bin Chi-squared values (in regular font) compare the expected and observed values for one subgenus in one soil bin. Values above 3.50 reflect a significant portion of the 5.99 critical value (CV) required for significance in the subgenus Chi square test. The subgenus Chi-squared tests (in bold) encompass all three soil bins for one subgenus (df = 2; p = 0.05*, CV = 5.99; p = 0.01**, CV = 9.21; p = 0.001***, CV = 13.82). The genus Chi-squared test combines Chi-squared values from both subgenera to indicate the attribute's overall influence on genus Thomomys (df = 4; p = 0.05*, CV = 9.49; p = 0.01**, CV = 13.28; p = 0.001***, CV = 18.47). (DOC) [file pone.0064935.s003.doc]

**Table S1.** Soil bin, subgenus and genus Chi square results for 6.5km dataset.

| **BULK DENSITY 20cm** | | | | | | | | |
| --- | --- | --- | --- | --- | --- | --- | --- | --- |
| *Soil Bin* | *Area (Km^2^)* | *% Area* | *Exp T.* | *Obs T.* | *Thom. X^2^* | *Exp M.* | *Obs M.* | *Meg. X^2^* |
| <1.1g/cm^3^ | 1.5 E+10 | 0.10 | 19 | 33 | 9.67 | 22 | 10 | 6.39 |
| 1.1-1.4 | 7.3 E+10 | 0.49 | 92 | 96 | 0.17 | 104 | 82 | 4.58 |
| >1.4 | 6.1 E+10 | 0.41 | 77 | 59 | 4.05 | 86 | 120 | 13.07 |
|  | 1.5 E+11 | 1 | 188 | 188 | **13.89***** | 212 | 212 | **24.03***** |
|  |  |  |  |  |  | **Genus *X^2^*:** | | **37.92***** |
| **BULK DENSITY 1m** | | | | | | | | |
| *Soil Bin* | *Area (Km^2^)* | *% Area* | *Exp T.* | *Obs T.* | *Thom. X^2^* | *Exp M.* | *Obs M.* | *Meg. X^2^* |
| <1.1g/cm^3^ | 1.1 E+10 | 0.07 | 14 | 28 | 15.18 | 15 | 3 | 9.95 |
| 1.1-1.4 | 6.9 E+10 | 0.47 | 87 | 102 | 2.42 | 99 | 90 | 0.75 |
| >1.4 | 6.9 E+10 | 0.46 | 87 | 58 | 9.63 | 98 | 119 | 4.48 |
|  | 1.5 E+11 | 1 | 188 | 188 | **27.24***** | 212 | 212 | **15.18***** |
|  |  |  |  |  |  | **Genus *X^2^*:** | | **42.41***** |
| **PERCENT CLAY 20cm** | | | | | | | | |
| *Soil Bin* | *Area (Km^2^)* | *% Area* | *Exp T.* | *Obs T.* | *Thom. X^2^* | *Exp M.* | *Obs M.* | *Meg. X^2^* |
| <20% | 8.3 E+10 | 0.56 | 105 | 130 | 6.21 | 118 | 94 | 4.84 |
| 20-30% | 5.4 E+10 | 0.36 | 68 | 44 | 8.73 | 77 | 106 | 10.76 |
| >30% | 1.2 E+10 | 0.08 | 15 | 14 | 0.07 | 17 | 12 | 1.45 |
|  | 1.5 E+11 | 1 | 188 | 188 | **15.00***** | 212 | 212 | **17.05***** |
|  |  |  |  |  |  | **Genus *X^2^*:** | | **32.05***** |
| **PERCENT CLAY 1m** | | | | | | | | |
| *Soil Bin* | *Area (Km^2^)* | *% Area* | *Exp T.* | *Obs T.* | *Thom. X^2^* | *Exp M.* | *Obs M.* | *Meg. X^2^* |
| <20% | 5.0 E+10 | 0.33 | 63 | 97 | 18.46 | 71 | 58 | 2.37 |
| 20-30% | 6.3 E+10 | 0.42 | 79 | 66 | 2.21 | 89 | 102 | 1.79 |
| >30% | 3.6 E+10 | 0.24 | 46 | 25 | 9.48 | 52 | 52 | 0.00 |
|  | 1.5 E+11 | 1 | 188 | 188 | **30.14***** | 212 | 212 | **4.16** |
|  |  |  |  |  |  | **Genus *X^2^*:** | | **34.30***** |
| **LINEAR EXTENSIBILITY 20cm** | | | | | | | | |
| *Soil Bin* | *Area (Km^2^)* | *% Area* | *Exp T.* | *Obs T.* | *Thom. X^2^* | *Exp M.* | *Obs M.* | *Meg. X^2^* |
| <1.5% | 5.3 E+10 | 0.35 | 67 | 90 | 8.29 | 75 | 42 | 14.52 |
| 1.5-3 | 6.4 E+10 | 0.43 | 81 | 66 | 2.70 | 91 | 108 | 3.15 |
| >3 | 3.2 E+10 | 0.22 | 41 | 32 | 1.87 | 46 | 62 | 5.63 |
|  | 1.5 E+11 | 1 | 188 | 188 | **12.86**** | 212 | 212 | **23.30***** |
|  |  |  |  |  |  | **Genus *X^2^*:** | | **36.16***** |
| **LINEAR EXTENSIBILITY 1m** | | | | | | | | |
| *Soil Bin* | *Area (Km^2^)* | *% Area* | *Exp T.* | *Obs T.* | *Thom. X^2^* | *Exp M.* | *Obs M.* | *Meg. X^2^* |
| <1.5% | 1.5 E+10 | 0.10 | 20 | 43 | 27.87 | 22 | 6 | 11.75 |
| 1.5-3 | 6.8 E+10 | 0.45 | 86 | 90 | 0.23 | 96 | 106 | 0.94 |
| >3 | 6.6 E+10 | 0.44 | 83 | 55 | 9.36 | 93 | 100 | 0.46 |
|  | 1.5 E+11 | 1 | 188 | 188 | **37.47***** | 212 | 212 | **13.16**** |
|  |  |  |  |  |  | **Genus *X^2^*:** | | **50.62***** |
| **DEPTH TO BEDROCK** | | | | | | | | |
| *Soil Bin* | *Area (Km^2^)* | *% Area* | *Exp T.* | *Obs T.* | *Thom. X^2^* | *Exp M.* | *Obs M.* | *Meg. X^2^* |
| 20-50cm | 2.6 E+10 | 0.18 | 31 | 24 | 1.66 | 37 | 37 | 0.00 |
| 50-1m | 3.0 E+10 | 0.15 | 26 | 32 | 1.55 | 30 | 23 | 1.77 |
| >1m | 9.6 E+10 | 0.67 | 115 | 116 | 0.01 | 136 | 143 | 0.38 |
|  | 1.5 E+11 | 1 | 172 | 172 | **3.22** | 203 | 203 | **2.14** |
|  |  |  |  |  |  | **Genus *X^2^*:** | | **5.37** |

Similar to Table 4 but using the 6.5km grid dataset. Soil bin Chi-squared values (in regular font) compare the expected and observed values for one subgenus in one soil bin. Values above 3.50 reflect a significant portion of the 5.99 critical value (CV) required for significance in the subgenus Chi square test. The subgenus Chi-squared tests (in bold) encompass all three soil bins for one subgenus (df = 2; p = 0.05*, CV = 5.99; p = 0.01**, CV = 9.21; p = 0.001***, CV = 13.82). The genus Chi-squared test combines Chi-squared values from both subgenera to indicate the attribute’s overall influence on genus *Thomomys* (df = 4; p = 0.05*, CV = 9.49; p = 0.01**, CV = 13.28; p = 0.001***, CV = 18.47).
